# Drought Stress Responses and Resistance in Plants: From Cellular Responses to Long-Distance Intercellular Communication

**DOI:** 10.3389/fpls.2020.556972

**Published:** 2020-09-10

**Authors:** Fuminori Takahashi, Takashi Kuromori, Kaoru Urano, Kazuko Yamaguchi-Shinozaki, Kazuo Shinozaki

**Affiliations:** ^1^Gene Discovery Research Group, RIKEN Center for Sustainable Resource Science, Tsukuba, Japan; ^2^Gene Discovery Research Group, RIKEN Center for Sustainable Resource Science, Wako, Japan; ^3^Laboratory of Plant Molecular Physiology, Graduate School of Agricultural and Life Sciences, The University of Tokyo, Bunkyo-ku, Japan

**Keywords:** drought stress, abscisic acid, peptides, transporters, protein kinases, metabolites, tissue-to-tissue communication

## Abstract

The drought stress responses of vascular plants are complex regulatory mechanisms because they include various physiological responses from signal perception under water deficit conditions to the acquisition of drought stress resistance at the whole-plant level. It is thought that plants first recognize water deficit conditions in roots and that several molecular signals then move from roots to shoots. Finally, a phytohormone, abscisic acid (ABA) is synthesized mainly in leaves. However, the detailed molecular mechanisms of stress sensors and the regulators that initiate ABA biosynthesis in response to drought stress conditions are still unclear. Another important issue is how plants adjust ABA propagation, stress-mediated gene expression and metabolite composition to acquire drought stress resistance in different tissues throughout the whole plant. In this review, we summarize recent advances in research on drought stress responses, focusing on long-distance signaling from roots to shoots, ABA synthesis and transport, and metabolic regulation in both cellular and whole-plant levels of Arabidopsis and crops. We also discuss coordinated mechanisms for acquiring drought stress adaptations and resistance *via* tissue-to-tissue communication and long-distance signaling.

## Introduction

Environmental stresses have multiple effects on plant growth. Extreme stresses often inflict severe damage during the production of plant biomass. Environmental stress conditions change either rapidly or incrementally. Therefore, plants must recognize and respond to stress conditions with a variety of biological signals at appropriate times and speeds for their survival (Takahashi and Shinozaki, 2019). Higher plants achieve sophisticated responses and adaptations to abiotic stresses, including drought, to maintain optimal growth under stress conditions. For these complex physiological responses in plants, a variety of cellular and molecular regulatory mechanisms are required for short-term responses to prevent water loss *via* transpiration from guard cells and for long-term adaptations to acquire stress resistance at the whole-plant level (Nakashima et al., 2014; Takahashi et al., 2018a).

Abscisic acid (ABA) is a key phytohormone that mediates drought stress responses and resistance by regulating stomatal closure and stress-responsive gene expression (Addicott et al., 1968; Cutler et al., 2010). In response to drought stress conditions, ABA accumulation is enhanced mainly in the vasculature of the leaves because almost all ABA biosynthesis enzymes are expressed in vascular tissues (Kuromori et al., 2018). In addition, several cellular-membrane-located ABA transporters are predominantly expressed at vascular tissues. Although it is not completely understood how ABA is regulated to move in tissue-to-tissue manner, the vasculature and apoplastic areas among tissues are described as the typical ABA route for the systemic signaling in classical textbooks. Therefore, accumulated ABA is supposed to spread from the vasculature to all tissues to mediate stomatal movements and gene expression related to drought stress resistance.

In general, the environmental conditions in the soil and atmosphere are completely different under drought stress conditions. Plants recognize the change of water deficit conditions in the soil appropriately under drought stress and transmit the water deficit signal from the roots to the leaves to adapt to drought stress conditions through ABA accumulation. Drought stress responses are also regulated by both ABA-dependent and ABA-independent regulatory systems (Nakashima et al., 2014; Takahashi et al., 2018a). Unlike animals, plants do not have a central nervous system, but their vascular system connects the roots and shoots and plays important roles in integrating stress information from the underground and aerial parts of plants. Many studies have reported that hydraulic signals, electric currents, calcium waves, reactive oxygen species (ROS), mRNAs, and phytohormone movements mediate drought stress responses (Christmann et al., 2013; Notaguchi and Okamoto, 2015; Choi et al., 2017; Kudla et al., 2018). Recent studies reported novel roles of hormone-like peptides as signaling molecules that mediate drought stress responses (Takahashi et al., 2018b; Takahashi et al., 2019). These novel findings suggest that peptides function as mobile molecules in the plant vasculature for the integration of water deficit stress signals in long-distance organ-to-organ communication. Recently, the regulatory mechanisms of both intracellular and intercellular responses have been analyzed in phytohormone signaling and transport, stress-mediated gene expression and metabolite production during drought stress responses.

In this review, we summarize recent knowledge of how long-distance signaling, ABA transport and metabolic regulation mediate drought stress responses and resistance in Arabidopsis and crops. These findings imply that plants have developed unique and complex mechanisms that connect various organs to resist environmental stresses and optimize growth. A comprehensive understanding of plant responses to drought stress will help to elucidate the mechanism by which local and long-distance responses are integrated at the whole-plant level.

## Long-Distance Communication in Drought Stress Responses and Resistance

### Dehydration Responses in Leaves *via* Root-to-Shoot Signaling

**Figure 1:**
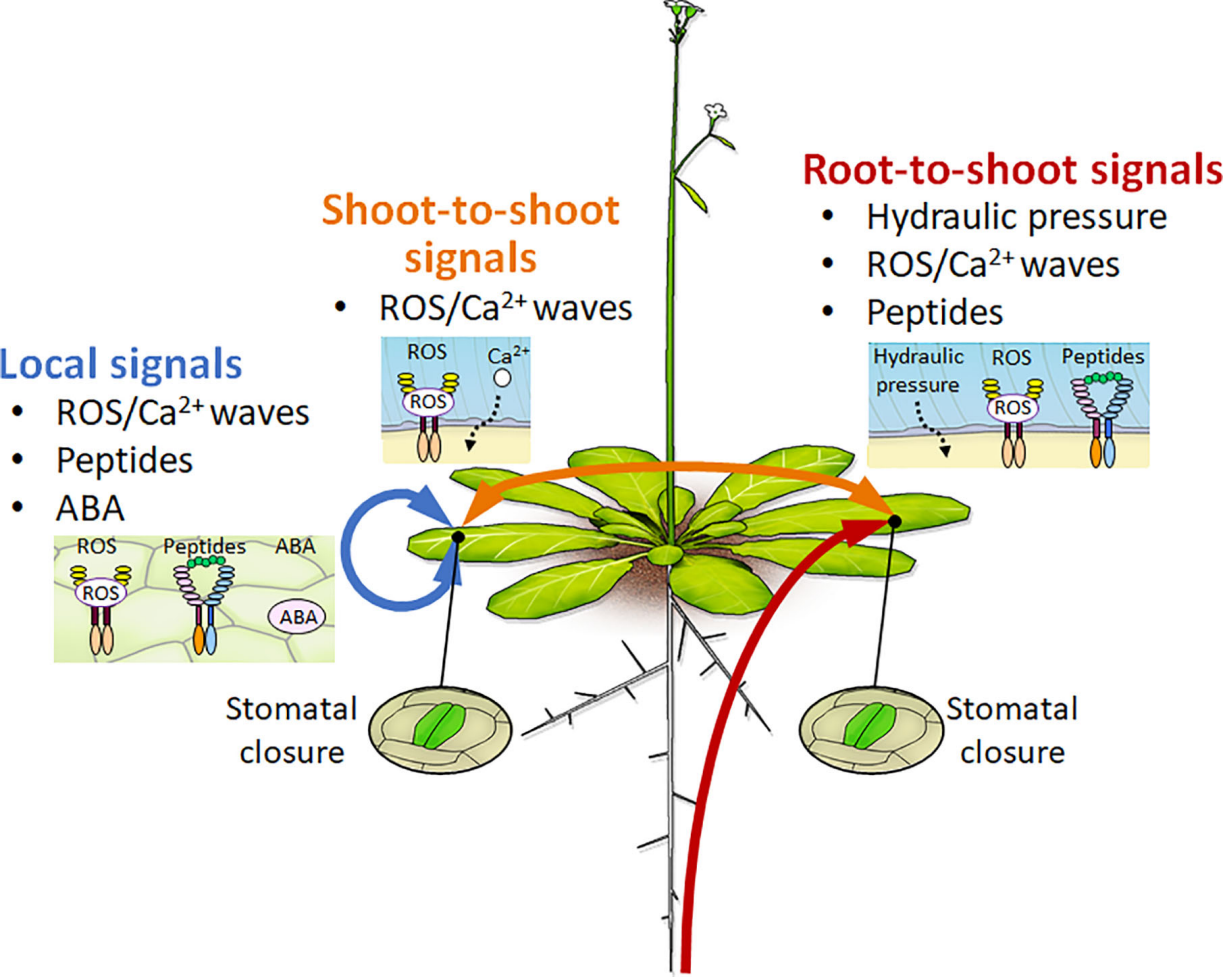
Conceptual diagram of root-to-shoot, shoot-to-shoot, and local signals in response to drought stress conditions. Mobile signals such as hydraulic pressure, ROS/Ca^2+^ waves, peptides, and phytohormones mediate tissue-to-tissue and long-distance communication for the acquisition of drought stress resistance at the whole-plant level. The red line indicates the root-to-shoot signals such as hydraulic pressure, ROS/Ca^2+^ waves, and peptides signals under dehydration stress conditions. The orange line indicates the shoot-to-shoot signals of ROS/Ca^2+^ waves to mediate stomatal closure under stress conditions. The blue line indicates the local signals of ROS/Ca^2+^ waves, peptide, or ABA signals that mediate stomatal control under stress conditions.

Vascular plants have developed some tissue-to-tissue communication systems by which various organs transmit the information of environmental stress conditions (**Figure 1**). As for one example, the decreasing water potential status is an initial step that plants recognize dehydration stress conditions (Christmann et al., 2013). Plants perceive water deficit status in their roots and integrate the information among distant organs. The hydraulic change signal can transmit through the vasculature at a speed of 1 m/min. Previous report indicates that lycophytes and ferns regulate stomatal closure in an ABA-independent manner (Brodribb and McAdam, 2011). *Vitis vinifera* regulates stomatal closure primarily in response to water deficit conditions, and then accumulates ABA to maintain the status of closed stomata (Tombesi et al., 2015).

On the other hand, previous studies indicated that dehydrated plants accumulate ABA in their bodies for regulating stomatal closure (Munemasa et al., 2015). The gene for a key enzyme in ABA biosynthesis, NINE CIS EPOXYCAROTENOID DIOXYGENASE3 (NCED3), is highly expressed in the vasculature of dehydrated leaves (Iuchi et al., 2001; Endo et al., 2008). Grafting experiments showed that the accumulation of ABA is more important in the leaves than in the roots for regulating stomatal closure (Holbrook et al., 2002; McAdam et al., 2016). In addition, the hydraulic stress caused by turgor loss activates ABA biosynthesis in the leaves (Christmann et al., 2007). Recently, the CLAVATA3/EMBRYO-SURROUNDING REGION-RELATED25 (CLE25) peptide was shown to function as a root-derived long-distance signal for ABA production in the leaves (Christmann and Grill, 2018; McLachlan et al., 2018; Takahashi et al., 2018b; Yoshida and Fernie, 2018). Cysteine produced from sulfate can mediate the stomatal closure through the accumulation of ABA and ROS (Batool et al., 2018). These results indicate that plants regulate stomatal aperture *via* various signals that are generated at different times during prolonged dehydration stress conditions (**Figure 1**). Further analyses are needed to understand how plants recognize water-deficit conditions at each organ. In addition, spaciotemporal analyses will reveal detailed stepwise responses for integrating complex dehydration stress information into long-distance communication at the cellular, organ, and whole-plant levels.

## CLE Peptides Mediate Drought Stress Responses in Long-Distance Signaling

Recent studies have suggested that some peptides mediate cell-to-cell and/or long-distance signaling to cover the phytohormones functions (Motomitsu et al., 2015; Notaguchi and Okamoto, 2015; Takahashi and Shinozaki, 2019). As a functional feature of these mobile peptides, the peptide-receptor modules in each tissue enable to transmit environmental stimuli precisely from the sensing tissues to the target tissues (**Figure 1**). Recent study reported that the CLE25 peptide modulates ABA biosynthesis to regulate stomatal closure during root-to-shoot signaling under dehydration stress conditions (Takahashi et al., 2018b). Root-derived CLE25 moves from the roots to the leaves and enhances ABA accumulation through the induction of *NCED3* expression in the leaves. BARELY ANY MERISTEM (BAM) 1 and BAM3 Receptor-like Protein Kinases (RLKs) perceive CLE25 in the leaves. Therefore, the CLE25–BAM1 and BAM3 systems control ABA accumulation and responses, including stomatal closure and stress-inducible gene expression. These results indicate that plants can integrate the water deficit information into their roots and leaves and optimize stress adaptations in those tissues.

The NGATHA1 (NGA1) transcription factor mediates dehydration stress-induced *NCED3* expression (Sato et al., 2018). The expression of *NGA1* gene is observed in the leaf vasculature. This indicates a physiological relationship between the CLE25–BAM1 and BAM3 signaling systems and the NGA1 in the regulation of *NCED3* expression.

*cle25* mutants show a normal root growth phenotype. On the other hand, overexpression of a partial dominant-negative mutant of CLE25 (with a G6T change in the amino acid sequence) inhibited differentiation of the protophloem sieve element (Ren et al., 2019). Systematic analyses revealed that almost all CLE peptide genes are expressed in roots (Jun et al., 2010). Exogenous applications of synthetic CLE peptides inhibit root growth elongation (Fiers et al., 2005; Ito et al., 2006; Kinoshita et al., 2007), indicating that several CLE peptides, including CLE25, redundantly mediate the regulation of root differentiation and development. It is generally known that plants retard shoot and root growth to save energy effectively under environmental stress conditions and acquire stress resistance. Taken together, these reports on CLE25 show that the function of CLE25 in the leaves and roots may be advantageous in adaptation to drought stress conditions in higher plants.

## Signaling Crosstalk for Stomatal Regulation

The acquisition of drought stress resistance occurs as a result of complex physiological signals, such as hydraulic, peptide, ABA, ROS, and calcium ion (Ca^2+^) current signals, in plants. ABA triggers an increase in Ca^2+^ currents through the activation of hydrogen peroxide (H_2_O_2_), which is a major component of ROS in guard cells (Hamilton et al., 2000; Pei et al., 2000).

Recently, HYDROGEN-PEROXIDE-INDUCED Ca^2+^ INCREASE (HPCA) was reported as a sensor of H_2_O_2_ in guard cells (Wu et al., 2020). HPCA1 belongs to subclass VIII-1 of Leucine-Rich-Repeat Receptor-Like Kinase (LRR-RLK), which is localized to the plasma membrane. The activation of HPCA1 occurs *via* the oxidization of two cysteine residue pairs that are present in the extracellular domain of HPCA1 by H_2_O_2_, which leads to the influx of Ca^2+^ currents in guard cells. As a result, *hpca1* mutants impair ABA- and H_2_O_2_-induced stomatal closure, indicating that HPCA1 is a key component of H_2_O_2_ signaling for stomatal control in guard cells.

Excessive light stress rapidly regulates stomatal closure through changes in photosynthesis and transpiration in plants. Light stress enhances ABA accumulation and then causes the generation of ROS/Ca^2+^ waves through the RESPIRATORY BURST OXIDASE HOMOLOG D (RBOHD). Both light stress-induced ABA and ROS/Ca^2+^ mediate stomatal closure in local leaves (Devireddy et al., 2018). The ROS/Ca^2+^ wave that is generated by light stress propagates rapidly from local leaves to systemic leaves at a speed of 100 μm/s and, in turn, mediates the initiation of ABA and jasmonic acid (JA) biosynthesis. ROS/Ca^2+^ wave-generated ABA and JA mediate stomatal closure with the activation of GUARD CELL HYDROGEN PEROXIDE-RESISTANT1 (GHR1) and the S-type anion channel SLAC1 in systemic leaves. Therefore, local ROS/Ca^2+^ waves function as long-distance signals to regulate ABA responses and stomatal closure in systemic leaves under (Devireddy et al., 2018; Yoshida and Fernie, 2018; Kollist et al., 2019) (**Figure 1**).

## Sensing Systems in Water Deficit Conditions

It is generally thought that membrane proteins, including RLKs, histidine kinases, and integrin-like proteins, function as osmotic stress sensors based on research in yeasts as well as in plants. *Arabidopsis thaliana* histidine kinase (ATHK) 1/*Arabidopsis* histidine kinase (AHK) 1 is localized at the plasma membrane of the cells and isolated as a homolog of the yeast two-component system in plants (Urao et al., 1999). ATHK1/AHK1 complements the function of SLN1 that encodes osmosensing histidine protein kinase in the yeast, indicating that ATHK1/AHK1 can acts as an osmotic stress sensor. Gain and loss of function of ATHK1/AHK1 showed that ATHK1/AHK1 mediates drought stress responses and resistance through ABA accumulation and drought stress-induced gene expression (Tran et al., 2007; Wohlbach et al., 2008). On the other hand, other T-DNA mutant lines of *athk1* did not show a significant difference in ABA accumulation compared to that of control plants under low water potential and instead displayed an increased density of stomata (Kumar et al., 2013). Compared to the control phenotype, the morphological phenotype of *athk1* mutants led to higher relative water loss from the leaves on soil under long-term drought stress conditions. The alteration of stomatal conductance caused by rapid changes in water potential did not mediate the *ATHK1/AHK1* mutation (Sussmilch et al., 2017). Other ATHK/AHKs, such as AHK2, AHK3, and AHK4, mediate osmotic stress responses negatively through cytokinin signaling (Jeon et al., 2010; Nishiyama et al., 2011). ATHK/AHKs are intricately linked to both stress responses and optimal growth under drought stress conditions. Further analyses including the isolation of molecules directly downstream of ATHK/AHKs will help us understand ATHK/AHK signaling in plants.

Calcium channels are also thought to be possible osmosensor candidates in the drought stress response. The Ca^2+^ uptake channels, Ca^2+^-permeable mechanosensitive channel (MCA) 1 and MCA2, mediate optimal growth under stress conditions (Nakagawa et al., 2007; Yamanaka et al., 2010). The MCA1 and MCA2 act as sensors of cell wall tension in plants because MCA1 and MCA2 functionally complemented the lethal phenotype of the *mid1* mutant in yeast. These results imply that MCA1 and MCA2 may mediate the perception of the change of hydraulic pressure caused by water potential under dehydration stress conditions.

## Local Signals That Mediate Dehydration Stress Responses and Stomatal Control

The guard cell–expressed *CLE9* gene was also reported to mediate dehydration stress responses (Zhang et al., 2019). Gain-of-function analyses indicated that CLE9 triggers both the activation of mitogen-activated protein kinase (MAPK) and the accumulation of ROS and enhances SLOW ANION CHANNEL ASSOCIATED1 (SLAC1) activity in guard cells to regulate stomatal closure. *cle9* mutants show the dehydration sensitive phenotype. The CLE9-induced stomatal closure is not inhibited in the *bam1* and *bam3* mutants, indicating that some other RLKs mediate the CLE9 signaling in guard cells.

REDUCED HYPEROSMOLALITY INDUCED Ca^2+^ INCREASE1 (OSCA1) mediates osmotic stress-induced Ca^2+^ influx in guard cells and root cells (Yuan et al., 2014). *osca1* mutants showed higher water loss than did control plants in response to osmotic stress treatment of the roots because the *osca1* mutant did not regulate stomatal closure under osmotic stress conditions. The detached leaves of *osca1* mutants also displayed enhanced water loss compared to that of control plants. On the other hand, ABA-induced stomatal closure occurred in the leaves of *osca1* mutants as well as control plants, indicating that OSCA1 mediates stomatal closure only with osmotic stress-induced Ca^2+^ influx in guard cells (**Figure 1**). CALCIUM-PERMEABLE STRESS-GATED CATION CHANNEL1 (CSC1)/OSCA1.2 is a homolog in the OSCA family and has been reported as a hyperosmolality-gated calcium-permeable channel protein (Hou et al., 2014). CSC1/OSCA1.2 also mediated calcium fluxes in plant cells in response to osmotic stress conditions, although the detailed function of CSC1/OSCA1.2 in plant tissues remains unclear (Liu et al., 2018; Maity et al., 2019).

As a matter of course, ROS and hydroxyl radicals, turgor pressure and Ca^2+^ also mediate stomatal regulation to prevent water loss through local and/or long-distance signaling under drought stress conditions (Christmann et al., 2013; Kollist et al., 2019). These results indicate that the mechanisms by which osmotic stress is perceived in plants may be complicated and that spatiotemporal responses precisely transmit information to tissues throughout the whole plant.

## ABA Transporters in Plants

ABA is a mobile signal in plants. Membrane transporters are important regulators of the intercellular networks of signaling molecules across cellular membranes. Certain kinds of membrane transporters have actually been reported as ABA membrane transporters in recent decades (Boursiac et al., 2013; Kuromori et al., 2018). Selected transporter families and protein members described in this section are listed in **Table 1**. Various membrane proteins function in ABA transport in different tissues, which indicates that complex systems are involved in ABA transport in plant physiology.

**Table 1:** Summary of membrane transporters reported as ABA transporters.

Gene family	Source organism	Gene name	Cellular localization	ABA transport direction	Ref.
ABC transporter	*Arabidopsis thaliana*	AtABCG25	PM	Exporter	Kuromori et al., 2010
	*Arabidopsis thaliana*	AtABCG40	PM	Importer	Kang et al., 2010
	*Arabidopsis thaliana*	AtABCG31	PM	Exporter	Kang et al., 2015
	*Arabidopsis thaliana*	AtABCG30	PM	Importer	Kang et al., 2015
	*Medicago truncatula*	MtABCG20	PM	Exporter	Pawela et al., 2019
	*Arachis hypogaea*	AhATL1	PM	(N.D.)	Ge et al., 20171150
	*Triticum aestivum*	Lr34	PM*	Importer	Krattinger et al., 2019 *Deppe et al., 2018
NPF transporter	*Arabidopsis thaliana*	AtNPF4.6	PM	Importer	Kanno et al., 2012
	*Medicago truncatula*	MtNPF6.8	PM	Importer (weak)	Pellizzaro et al., 2014
MATE transporter	*Arabidopsis thaliana*	AtDTX50	PM	Exporter	Zhang et al., 2014
	*Arabidopsis thaliana*	AtMATE45	TGN/Golgi	Importer (weak)	Kovinich et al., 2018
Other transporter	*Oryza sativa*	OsPM1	PM	Importer	Yao et al., 2018

ABC, ATP-binding cassette subfamily; NPF, nitrate transporter 1/peptide transporter family; MATE, multidrug and toxin efflux, DTX: detoxification efflux carriers; PM, plasma membrane; TGN, trans-Golgi network; N.D., not detectable.

*The cellular localization of Lr34 is described in the second reference, Deppe et al. (2018).

## ABC Transporter Family

Several ABA transporters reported to date belong to the ATP-binding cassette (ABC) transporter family. ABC transporters are highly conserved in most organisms, and terrestrial plants have two to four times more ABC transporter genes in their genomes than do animals, suggesting that some ABC transporters may have plant-specific functions in development and physiological regulation (Hwang et al., 2016; Do et al., 2018). Soon after the discovery that cytosolic ABA receptors are located inside of cells in *Arabidopsis thaliana* (Ma et al., 2009; Park et al., 2009), two ABC transporters, *AtABCG25* and *AtABCG40*, were identified as ABA transporters (Kang et al., 2010; Kuromori et al., 2010). Both of them were originally found in *Arabidopsis* tagged-mutant screening approaches based on ABA-related phenotypes. The *AtABCG25* promoter was predominantly active in the vascular tissue, and the *AtABCG40* promoter had the highest expression in guard cells. Both AtABCG25 and AtABCG40 localized to the plasma membrane when expressed as fluorescent fusion proteins in plant cells. An additional study showed that plasma membrane localization of AtABCG25 was regulated by abiotic stress and ABA (Park et al., 2016). Experimental assays of the ABA transport activity of each transporter finally revealed that *AtABCG25* mediates ABA export from the cell and *AtABCG40* mediates ABA import into the cell. This is consistent with distant ABA mobility and supports the hypothesis that ABA may serve as a mobile signal between vascular tissues and epidermal tissues, including guard cells, to control stomatal appearance (Kuromori and Shinozaki, 2010; Kuromori et al., 2014). AtABCG22 is a closely related member of the same subfamily that was reported to be involved in stomatal closure in *Arabidopsis*. *AtABCG22* is strongly expressed in guard cells. However, its transporter activity has not yet been elucidated (Kuromori et al., 2011).

In addition to stomatal regulation, ABA also substantially controls germination arrest in seeds. *AtABCG31* and *AtABCG30*, as well as *AtABCG25* and *AtABCG40* described above, were shown to serve as ABA exporters and importers, respectively, in *Arabidopsis* seeds (Kang et al., 2015). In mature seeds, ABA is released from the endosperm toward the embryo to repress seed germination (Lee et al., 2012). AtABCG31 was expressed at high levels and AtABCG25 was mainly expressed in the endosperm; in contrast, AtABCG30 and AtABCG40 were strongly expressed in dissected embryos. Additionally, plasma membrane localization of GFP-fusion proteins of AtABCG31 and AtABCG30 was shown in *Arabidopsis* mesophyll protoplasts. The absence of these transporters in knockout mutants resulted in disruption of the ABA distribution within seeds and a shortened germination time. These four ABC transporters act in concert to deliver ABA from the endosperm to the embryo to control germination in *Arabidopsis*; AtABCG25 and AtABCG31 export ABA from the endosperm, whereas AtABCG30 and AtABCG40 import ABA into the embryo (Kang et al., 2015).

More recently, the identification of ABA membrane transporters in the ABC transporter family has been extended to other plant species beyond the model plant *Arabidopsis thaliana*. For example, in *Medicago truncatula*, MtABCG20 was reported as an ABA transporter (Pawela et al., 2019). This gene was initially identified as strongly inducible under drought stress-mimicking conditions or ABA treatment. MtABCG20 was expressed at the hypocotyl-radicle transition zone in seeds and along vascular bundles, lateral root (LR) primordium sites and nodules in roots. Consistent with these expression sites, *mtabcg20* mutants were more sensitive to ABA upon germination of seeds. Furthermore, the mutants had two root phenotypes: fewer LRs and more nodules than wild-type roots under drought stress-mimicking conditions. While ABA signaling largely inhibits LR development in *Arabidopsis*, ABA primarily plays a positive role in LR development in *Medicago truncatula* (Gonzalez et al., 2015). Nodulation is a unique process in legumes, and ABA acts as a negative regulator of nodule primordium formation in the root cortex tissue (Ding et al., 2008). The *mtabcg20* mutant phenotypes might suggest that ABA accumulates at its place of biosynthesis as a consequence of impaired ABA transport. Actually, ABA export activity of MtABCG20 was shown by a transport assay using MtABCG20-overexpressing *Nicotiana tabacum* BY2 cells. Additionally, MtABCG20 showed subcellular localization at the plasma membrane *via* homodimer formation in *Arabidopsis* mesophyll protoplasts. Together, these data indicate that MtABCG20 is an ABA transporter that influences root morphology and nodulation in *Medicago truncatula* (Pawela et al., 2019).

In legumes, another ABC transporter encoded by the *Arachis hypogaea* ABA transporter-like 1 (AhATL1) gene has been reported as a potential ABA transporter (Ge et al., 2017). In peanut plants, AhATL1 was upregulated by water stress and exogenous ABA treatment. Overexpression of AhATL1 in *Arabidopsis* decreased ABA sensitivity and drought resistance by inhibiting drought-induced AtABCG40 expression in guard cells. While the ABA transport activity of AhATL1 has not yet been shown, the function of AhATL1 was postulated to modulate ABA sensitivity by specifically influencing ABA import into cells (Ge et al., 2017).

An ABA membrane transporter in the ABC transporter family has also been reported in monocot plants, including important agricultural crops. For example, in *Triticum aestivum*, Lr34 was identified as an ABA transporter (Hulbert et al., 2007). This factor was initially identified as a gene that confers durable adult plant resistance against multiple fungal diseases (Ellis et al., 2014). In the wheat gene pool, the Lr34 gene was present in two different alleles, called Lr34sus and Lr34res, which were characterized by their susceptible and resistant phenotypes, respectively, to biotrophic fungal pathogens. Lr34res-responsive genes in wheat have been associated with ABA inducibility (Hulbert et al., 2007). In addition, in Lr34res-expressing transgenic rice plants, Lr34res induced transcripts reminiscent of an ABA-regulated stress response. Lr34res-expressing transgenic rice plants also showed alterations in biological processes that are controlled by ABA (Hulbert et al., 2007). In transgenic rice seedlings, increased ABA accumulation was specific to the Lr34res-expressing lines, which correlated with the induction of ABA-regulated genes, physiological alterations, and disease resistance. *In vitro* yeast accumulation assays demonstrated that the Lr34res protein resulted in increased ABA uptake in yeast cells, suggesting that LR34res might act as an ABA importer. Because of its durability and broad-spectrum specificity, the *Lr34* gene has been applied as one of the most frequently used disease resistance genes in wheat breeding. Moreover, Lr34res has been shown to be functionally transferrable as a transgene in all major cereals, including barley, rice, maize, and sorghum (Hulbert et al., 2007).

## NPF, MATE, and Other Transporter Families

In addition to the ABC transporter family, several ABA transporters in different families have been reported thus far. For example, *Arabidopsis* NPF4.6, which was originally named ABA-IMPORTING TRANSPORTER1 (AIT1), and several members of the NITRATE TRANSPORTER 1/PEPTIDE TRANSPORTER FAMILY (NPF) have been identified as ABA transporters (Kanno et al., 2012; Chiba et al., 2015; Tal et al., 2016; Leran et al., 2020). NPF4.6 and related family members were successfully identified using a modified yeast two-hybrid system screen of *Arabidopsis* cDNAs to identify proteins capable of inducing interactions between the ABA receptor PYR/PYL/RCAR and the PP2C protein phosphatase under low ABA concentrations in yeast cells. Overall, transport assays in insect cells expressing NPF4.6 demonstrated that this transporter mediated cellular ABA uptake into cultured cells (Kanno et al., 2012). Fluorescent protein-fused NPF4.6 proteins were detected predominantly at the plasma membrane. Less sensitivity to exogenously applied ABA during seed germination and initial growth in seedlings was observed in *npf4.6* mutants than in wild-type plants. In contrast, overexpression of NPF4.6 resulted in ABA hypersensitivity in seeds and during the initial stages of growth. In adult plants, the inflorescence stems of *npf4.6* mutants had a lower surface temperature than did those of wild-type plants. NPF4.6 promoter activity was detected in imbibed seeds and vascular tissues of cotyledons, true leaves, hypocotyls, roots, and inflorescence stems using a promoter-reporter system. These data suggest that the function of NPF4.6 as an ABA importer in vascular regions affects the regulation of stomatal aperture in shoots (Kanno et al., 2012).

NPF transporters have been functionally characterized as nitrate transporters, but many members may also act as dual-affinity transporters (Chiba et al., 2015). One of the NPF members of *Medicago truncatula*, MtNPF6.8, has been reported to be involved in nitrate-mediated inhibition of primary root growth in a manner dependent on ABA signaling (Pellizzaro et al., 2014). In addition to nitrate influx activity, MtNPF6.8 was detected to have ABA uptake activity, although at a low rate, in the *Xenopus* oocyte assay, suggesting an additional role in ABA translocation as well as nitrate relocation (Pellizzaro et al., 2014).

In another transporter family, *Arabidopsis thaliana* DETOXIFICATION EFFLUX CARRIER 50 (AtDTX50), which belongs to the multidrug and toxin efflux transporter (MATE) family, has also been identified as an ABA transporter in *Arabidopsis* (Zhang et al., 2014). This gene was first found in a comprehensive reverse genetic study of MATE transporters in *Arabidopsis*. A mutant defective in AtDTX50 exhibited growth defects, possibly due to its higher sensitivity to ABA or higher endogenous ABA levels. Fluorescence-tagged AtDTX50 proteins were localized predominantly to the plasma membrane, and AtDTX50 facilitated ABA efflux from inside to outside of cells in *Escherichia coli* and *Xenopus* oocytes. AtDTX50 was preferentially expressed in vascular tissues, indicating that it might regulate ABA export from vascular tissues in *Arabidopsis*, possibly in association with AtABCG25 and NPF4.6 because of their similar expression sites. On the other hand, AtDTX50 was also expressed in guard cells, and the *atdtx50* mutant plants were more tolerant to drought, with lower stomatal conductance. These data indicate that AtDTX50 might modify ABA transport in both vascular cells and guard cells (Zhang et al., 2014).

In the *Arabidopsis* MATE transporter family, AtMATE45 has been reported as a candidate ABA transporter (Kovinich et al., 2018). AtMATE45 was first identified as one of four transporter genes found by genetic screening to observe anthocyanin pigmentation under anthocyanin induction conditions (AICs). Anthocyanin pigmentation related to ABA levels and ABA signaling has been demonstrated (Adams et al., 1999). Related to another ABA transporter described above in the ABC family, AtABCG25 was also included among four transporter genes found by genetic screening in AICs (Kovinich et al., 2018). AtMATE45 is mostly expressed at growing meristem sites and in the vasculature in *Arabidopsis*. The *atmate45* mutant exhibited delayed germination of seeds, reduced rosette biomass, increased numbers of inflorescence stems, and shorter siliques. These phenotypes were ABA-dependent but not due to the anthocyanin level, as shown by the double-mutant analysis. The observation of GFP-fusion proteins with subcellular markers and BFA treatment, which blocks vesicle trafficking, revealed that AtMATE45 was localized to the subcellular trans-Golgi network (TGN) and Golgi apparatus. The subcellular localization of AtMATE45 is extremely unique because every other ABA transporter referred to in this article is a plasma membrane protein. Slight but significant ABA uptake activity of AtMATE45 was detected in *E. coli* cells expressing AtMATE45. These results suggest that AtMATE45 may antagonize meristematic ABA signaling, although it is unlikely to function as a bona fide ABA transporter (Kovinich et al., 2018).

Additionally, a novel type of membrane protein has been reported as a rice ABA transporter (Yao et al., 2018). *Oryza sativa* PLASMA MEMBRANE PROTEIN 1 (OsPM1) belongs to a protein family named ABA-induced Wheat Plasma Membrane polypeptide-19 (AWPM-19), whose functions were previously unknown. *OsPM1* showed strong induction in response to drought in all developmental stages. The promoter-GUS transgenic rice showed that the gene was expressed weakly in vascular tissues, guard cells, and mature embryos under normal conditions and strongly induced by ABA at all these sites. The *ospm1* knockout mutants and knockdown RNAi lines showed greater drought sensitivity than did wild-type plants. By contrast, the OsPM1-overexpression lines showed an obvious drought resistance phenotype, and their survival rates were much higher than those of wild-type plants under water stress conditions, probably because OsPM1 might be involved in ABA-induced stomatal closure. Furthermore, many ABA-responsive genes were upregulated more in the OsPM1-overexpression lines but less in the RNAi lines compared to wild-type plants (Yao et al., 2018). The YFP-OsPM1 fusion proteins clearly appeared in the plasma membrane portion but not in the cytoplasm. The yeast cells expressing OsPM1 accumulated more ABA than the control cells, and ABA substrate specificity and pH dependency were observed. Consistently, a FRET sensor plasmid (Jones et al., 2014) revealed that yeast cells expressing OsPM1 showed more ABA uptake due to stronger induced fluorescence changes than cells transformed with the empty vector control (Yao et al., 2018). All these data indicate that OsPM1 has a physiological function as an ABA influx transporter and plays a significant role in drought responses.

## Metabolic Regulation Mediating Drought Stress Responses and Resistance

**Figure 2:**
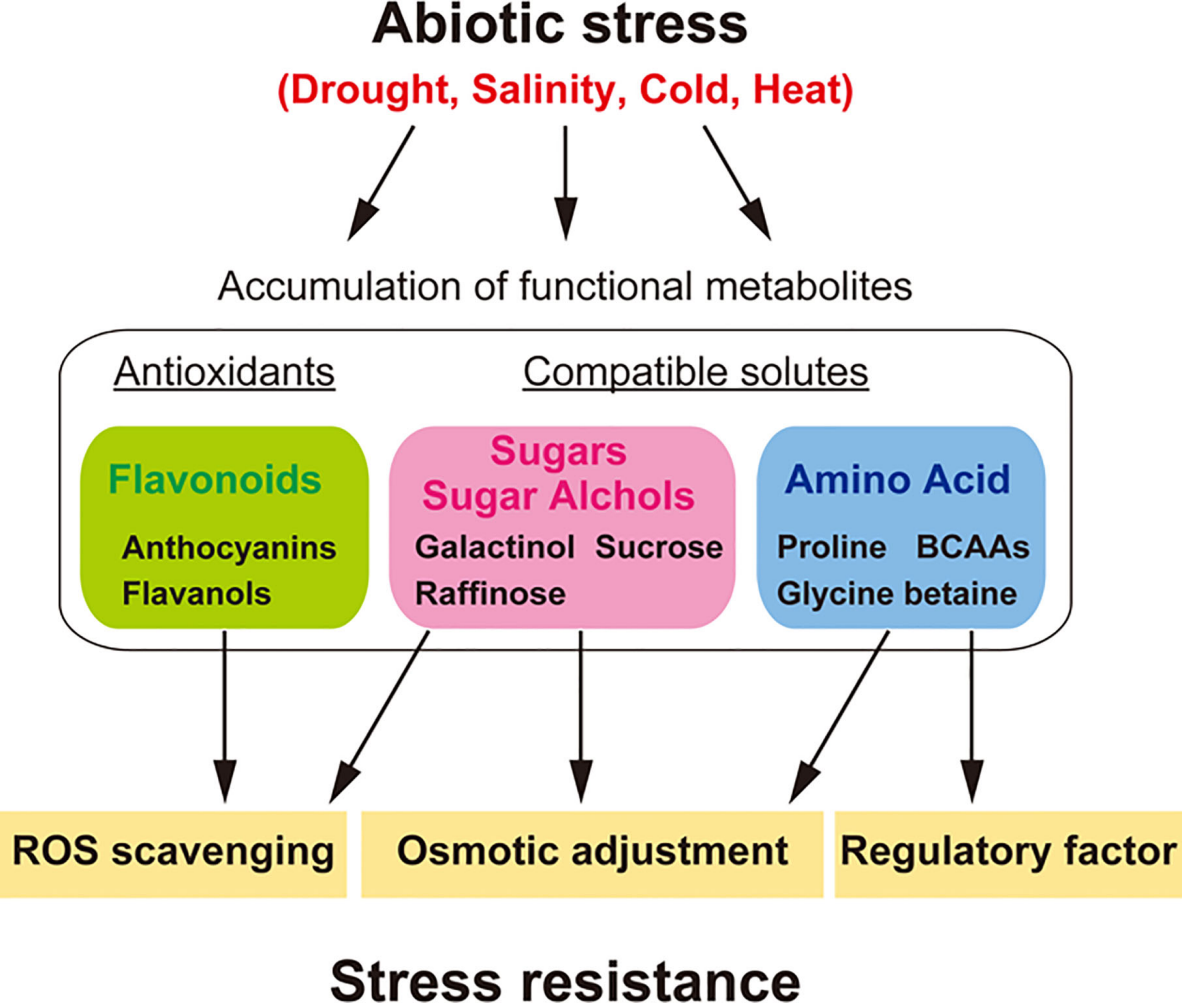
Metabolites and their functions in abiotic stress tolerance, especially under drought, salinity, cold and heat stress. Plants show a variety of metabolic responses to diverse abiotic stresses. Stress-induced accumulation of compatible solutes such as sugars (e.g., raffinose and sucrose), sugar alcohols (e.g., galactinol), and amino acids (e.g., BCAAs, proline, and glycine-betaine) functions in osmotic adjustment, enabling the maintenance of cell turgor for plant growth and survival under stress conditions. These compatible solutes, especially galactinol and raffinose, can act as free radical (ROS) scavengers, protecting against oxidation by removing excess ROS and reestablishing the cellular redox balance. BCAAs can act as regulatory factors in the production of specialized/secondary metabolites as a defense response against biotic stress during abiotic stress. Stress-induced accumulation of antioxidants such as flavonoids (e.g., anthocyanins and flavonols) allows them to act as free radical scavengers to mitigate oxidative and drought stress in plants. Genes involved in metabolite synthesis in diverse abiotic stress responses are useful for application in the metabolic engineering of stress resistance in dry field conditions.

The metabolic profiles of plants change in response to environmental stress factors such as drought, high salinity, and temperature (Urano et al., 2010; Cramer et al., 2011) (**Figure 2**). Metabolic profiles have been analyzed to study the function of the plant environmental stress response and tolerance (Levitt, 1972; Bartels and Sunkar, 2005; Schauer and Fernie, 2006; Sweetlove et al., 2008). It has been proposed that soluble sugars and other charged metabolites, such as proline and glycine-betaine, accumulate throughout the plant body, and function as compatible solutes during stress responses. Stress-induced accumulation of these metabolites lowers the water potential of the cell, promoting water retention in the plant without interfering with normal metabolism. This process, known as osmotic adjustment, enables the maintenance of cell turgor for plant growth and survival under stress conditions (Bartels and Sunkar, 2005; Verslues and Sharma, 2010; Krasensky and Jonak, 2012). In addition, these compatible solutes stabilize proteins and cell structures, particularly when osmotic stress becomes severe or persists for longer periods (Hoekstra et al., 2001). These metabolites also act as free radical scavengers, protecting against oxidation by removing excess ROS and reestablishing the cellular redox balance (Couee et al., 2006; Miller et al., 2010). On the other hand, specialized/secondary metabolites such as flavonoids have defense functions against both biotic and abiotic stress (Dixon and Paiva, 1995). Major flavonoids, such as flavonols (kaempferols and quercetins) and anthocyanins (cyanidins), increase in response to drought stress to enhance stress tolerance (Nakabayashi et al., 2014a; Nakabayashi et al., 2014b). These specialized compounds also act as free radical scavengers to mitigate oxidative and drought stress in plants (**Figure 2**).

Since 1998, the development of metabolomics technologies using highly sensitive mass spectrometry (MS), nuclear magnetic resonance (NMR), and bioinformatics has enhanced the comprehensive analysis of diverse plant metabolites and the changes in their profiles that occur in response to environmental stress factors (Rizhsky et al., 2002; Cook et al., 2004; Hirai et al., 2004; Kaplan et al., 2004; Gong et al., 2005; Kaplan et al., 2007; Brautigam et al., 2009; Maruyama et al., 2009; Tschoep et al., 2009; Urano et al., 2009; Wulff-Zottele et al., 2010; Caldana et al., 2011; Kusano et al., 2011; Krasensky and Jonak, 2012). Integrated analysis of transcriptome and metabolome analyses in Arabidopsis is important not only for the comprehensive analysis of metabolic networks but also for the analyses of specific regulatory networks in stress-related metabolism, especially ABA-dependent and independent pathways (Urano et al., 2009; Urano et al., 2010; Cramer et al., 2011). Here, we focus on recent progress in the characterization of metabolic responses in environmental stress and the application of metabolic approaches for drought-tolerant crops in the field.

## Integrated Metabolomics Reveals the ABA-Dependent and ABA-Independent Metabolic Networks in Response to Abiotic Stress

Integrated analyses of the transcriptome and the metabolome in Arabidopsis successfully demonstrated connections between genes and primary metabolites, elucidating a wide range of signal outputs from ABA under dehydration (Urano et al., 2009). Metabolite profiling by GC/MS and CE/MS analyses revealed that ABA accumulates during dehydration, regulating the accumulation of various amino acids and sugars such as glucose and fructose. In particular, the dehydration-inducible accumulation of branched-chain amino acids (BCAAs), saccharopine, proline, and agmatine is correlated with the dehydration-inducible expression of their key biosynthetic genes (*branch-chain aminotransferase 2*; *AtBCAT2*, *lysine ketoglutarate reductase/saccharopine dehydrogenase*; *AtLKR/SDH*, delta 1-pyrroline-5-carboxylase 1; *AtP5CS1*, and *arginine decarboxylase 2*; and *AtADC2*, respectively), which are regulated by endogenous ABA (Urano et al., 2009).

In addition, analyses of the metabolic profiles of mutants of core regulators of ABA signaling, bZIP transcription factor ABA-RESPONSIVE ELEMENT BINDING PROTEIN/ABA-RESPONSIVE ELEMENT BINDING FACTOR (*AREB/ABF*) and subclass III SNF1-related protein kinase 2 (*SnRK2s*), could provide more detailed and significant information about the ABA-regulated metabolome in the context of plant stress tolerance and growth (Thalmann et al., 2016; Yoshida et al., 2019). The metabolic profile of starch degradation in mutants for ABA biosynthesis and signaling revealed that the ABA-subclass III SnRK2-AREB/ABF signaling pathway is important for the transcriptional regulation of *α-AMYLASE3* and *β-AMYLASE1* to release sugars and sugar-derived compatible solutes from starch degradation in response to osmotic stress (Thalmann et al., 2016). The subclass III SnRK2 signaling pathway is important for the modulation of organic acid and amino acid metabolism and leaf growth under nonstress conditions by fine-tuning flux through the tricarboxylic acid (TCA) cycle (Yoshida et al., 2019).

On the other hand, metabolome analysis of transgenic Arabidopsis overexpressing *AtDREB1A/CBF3* revealed an ABA-independent metabolic network that is strikingly similar to the low-temperature regulated metabolome (monosaccharides, disaccharides, oligosaccharides, and sugar alcohols) and is regulated by the transcription factor *AtDREB1A/CBF3* (Cook et al., 2004; Maruyama et al., 2009). In particular, the low-temperature-inducible accumulation of galactinol and raffinose is correlated with the expression of the *galactinol synthase 3* (*AtGols3*) gene, which is a direct target of *AtDREB1A/CBF3* (Cook et al., 2004; Maruyama et al., 2009). Maruyama et al. also analyzed *AtDREB2A* overexpression, which did not increase the level of any low-temperature regulated metabolites in transgenic plants. Overexpression of *AtDREB2A* in transgenic plants increased their tolerance to dehydration stress but only slightly increased their tolerance to freezing stress (Sakuma et al., 2006). These results indicate that the increased tolerance to freezing stress in transgenic plants overexpressing *AtDREB1A* may depend on the accumulation of low-temperature regulated metabolites, especially sucrose, raffinose, galactinol, and myo-inositol. Similarly, transcriptomics and metabolomics analyses of the *PSEUDO RESPONSE REGULATOR* (*PRR*) arrhythmic triple mutant revealed that the *AtDREB1A/CBF* gene and raffinose accumulation are regulated by the circadian clock in anticipation of colder night temperatures (Fukushima et al., 2009).

## Comprehensive Overview of Metabolic Profiles in Environmental Stress Responses

Metabolic profiling of Arabidopsis under various environmental conditions, such as dehydration, salinity, heat, cold, light, and nutrient limitation, enables us to both obtain a comprehensive overview of the metabolic network and understand crucial components involved in the response to abiotic stress. The metabolites that respond to various stresses can act as compatible solutes and play a role in fundamental abiotic stress tolerance. A stress-specific response at the metabolite level can be the result of transient inhibition or activation of a specific metabolic pathway due to the properties of enzymes under stress conditions, such as temperature, oxidation, and ion concentrations (Obata and Fernie, 2012). As expected, some metabolites, such as pyruvate- (alanine, isoleucine, leucine, and valine), oxaloacetate- (asparagine, lysine, methionine, and threonine), amine-containing metabolites (*β*-alanine, GABA, and putrescine), and carbohydrates (galactinol, maltose, sucrose, trehalose, and raffinose), were reported to increase in response to various abiotic stresses in metabolome analyses (Rizhsky et al., 2002; Cook et al., 2004; Hirai et al., 2004; Kaplan et al., 2004; Gong et al., 2005; Kaplan et al., 2007; Brautigam et al., 2009; Maruyama et al., 2009; Tschoep et al., 2009; Urano et al., 2009; Wulff-Zottele et al., 2010; Caldana et al., 2011; Kusano et al., 2011; Krasensky and Jonak, 2012). Metabolome analyses showed that a variety of primary metabolites act collectively as compatible solutes. They are very soluble in water and nontoxic at high concentrations, and they function to sustain the ordered vicinal water around proteins by decreasing protein-solvent interactions at low water activities (Bartels and Sunkar, 2005). The combination of compatible solutes exerts additive or synergistic effects under abiotic stress. In addition, these metabolites act as not only compatible solutes but also signaling molecules, antioxidants, or defenses against pathogens (Kaplan et al., 2004).

Although proline and carbohydrates have been characterized as functional metabolites under osmotic stress (Yoshiba et al., 1995; Nanjo et al., 1999; Taji et al., 2002; Li et al., 2011), metabolomics studies have contributed to the identification of novel groups of metabolites that generally accumulate in response to various stress conditions. In particular, BCAAs, such as isoleucine, leucine, and valine, were found to show ABA-dependent regulation at the transcript level under dehydration stress (Urano et al., 2009) and to function as compatible solutes due to their high fold increases in various plant tissues (Joshi et al., 2010; Obata and Fernie, 2012). BCAAs also serve as precursors for cyanogenic glycosides (Vetter, 2000). The accumulation of BCAAs supports the increased production of secondary metabolites as a defense response against biotic stress during abiotic stress. Such stress responses function as preemptive defense responses against pathogen attack on stressed host plants (**Figure 2**).

Proline is known as a major compatible solute in various plants under environmental stresses (Bartels and Sunkar, 2005; Verslues and Sharma, 2010; Krasensky and Jonak, 2012). However, comparison of various metabolome analyses under abiotic stress revealed that proline has been reported in a limited number of studies on plant abiotic stress responses (Obata and Fernie, 2012). Therefore, proline is thought to mainly function in response to osmotic stress. This was supported by the expression of key genes for proline biosynthesis in Arabidopsis, delta 1-*AtP5CS1* and *AtP5CS2*. Although *AtP5CSs* are induced by cold, dehydration, mannitol, and salt treatments (Yoshiba et al., 1995; Knight et al., 1997; Urano et al., 2009), they are not altered by heat, methyl viologen, and UV-B treatments in the Arabidopsis eFP Browser (https://bar.utoronto.ca/efp/cgi-bin/efpWeb.cgi) (Winter et al., 2007). Several studies have suggested that the intermediate of proline biosynthesis, delta 1-pyrroline-5-carboxylate (P5C), is toxic to plant cells (Hellmann et al., 2000; Deuschle et al., 2001; Mani et al., 2002). Because proline biosynthesis and degradation occur in mitochondria, the accumulation of toxic compounds such as P5C may cause damage to plant cells under severe abiotic stress (Rizhsky et al., 2002; Rizhsky et al., 2004). Plants are subjected to complex abiotic stress conditions in their natural habitats. Metabolite profiling of Arabidopsis responses to combined dehydration and heat stress (Rizhsky et al., 2004) has revealed that proline shows the highest accumulation among all metabolites under dehydration stress. On the other hand, sucrose showed the highest accumulation under combined dehydration and heat stress. These results revealed that sucrose replaces proline in plants as the major compatible solute during severe abiotic stress with both osmotic and oxidative damage. Sucrose, glucose, and fructose contents are distributed throughout the plant body upon exposure to drought stress in soybean (Maruyama et al., 2020). In contrast, other sugars and related polyol, raffinose, galactinol, and trehalose were highly accumulated in roots compared with those in shoots under drought stress. Pinitol and ononitol that are polyols detected in legume were specifically accumulated in drought-treated leaves of soybean. Distribution of sugars in plant is altered in response to drought stress, and function as a compatible solute in various organs. In additions, sugars such as sucrose, glucose, and trehalose also act as signal molecules to regulate gene expression in plant growth and stress response (Maruyama et al., 2014). To improve plant tolerance under field conditions, enhancement of the synthetic pathways of metabolites such as raffinose and/or sucrose is thought to be useful for metabolic engineering of drought tolerance, as shown in the next section.

## Metabolic Engineering Approaches Have Contributed to the Development of Stress-Tolerant Crops in the Field

The development of drought-tolerant crops seems to be a promising solution to increase crop yield under water-limited conditions to fulfill the food requirements of increasing world populations (Soni et al., 2015). Drought stress has often caused significant decreases in crop production, which is caused by continuous global warming. Enhancing drought tolerance without a grain yield penalty has been a great challenge in crop improvement. Many genes have been tested in greenhouses, but few of them have proven to be useful in the field. Due to the complexity of stress conditions in the field, the enhancement of stress-related metabolites with no effect on plant growth seems to be a good strategy for the metabolic engineering of drought tolerance.

Recently, engineering of the raffinose biosynthetic pathway was shown to be successful in breeding drought stress tolerance in dry field conditions (Honna et al., 2016; Selvaraj et al., 2017). Arabidopsis genes for raffinose biosynthesis were identified by Taji et al., 2002. *Galactinol synthase* (*AtGolS*) is a key gene for galactinol and raffinose accumulation under drought, heat, and cold stress conditions. Among them, the *AtGolS*2 gene is specifically induced by drought stress. The expression of *AtGolS2* was also increased under oxidative stress and regulated by heat-shock transcription factor A2 (*AtHsfA2*) (Nishizawa et al., 2008). Overexpression of *AtGolS2* in transgenic Arabidopsis and Brachypodium caused an increase in galactinol and raffinose levels, improved drought stress tolerance (Taji et al., 2002; Himuro et al., 2014) and protected plants from oxidative stress (Nishizawa et al., 2008). These findings support that galactinol and raffinose have a strong ability to protect cells as compatible solutes and ROS scavengers under several types of environmental stresses, such as dry field conditions. Metabolome analyses in rice and soybean revealed that galactinol and raffinose levels were increased in response to dehydration stress (Maruyama et al., 2014; Maruyama et al., 2020). The application of *AtGolS2* for rice and soybean transformation has been attempted to validate stress responses and phenotypes under dry field conditions (Honna et al., 2016; Selvaraj et al., 2017). Overexpression of *AtGolS2* in transgenic rice and soybean not only improved drought tolerance but also increased grain yield in dry field conditions (Honna et al., 2016; Selvaraj et al., 2017). These reports showed that metabolic engineering of *AtGolS2* is a useful biotechnological tool to reduce grain yield losses under drought stress in the field (**Figure 2**).

## Conclusions and Future Perspectives

Plants have evolved to respond and adapt properly to drought stress conditions through spatiotemporal and stepwise signals in tissues throughout the whole plant to acquire drought stress resistance. The phytohormone ABA regulates drought stress responses and resistance at the cellular and intercellular levels in plants. However, it remains unclear how plants perceive drought stress conditions and transmit that information into the cell to regulate ABA accumulation for the acquisition of drought stress resistance. Regulatory factors involved in tissue-to-tissue communication and long-distance signaling have been focused recently because these mobile molecules complement the movement of ABA from the synthesis tissues to the target tissues. There are ABA receptors in almost cells. On the other hand, the receptors that bind with peptides are expressed specifically in each tissue. These diverse expressions allow the peptide-receptor modules to transmit the information of environmental conditions accurately from the sensing tissues to the target tissues than does the ABA regulatory system. Advances in molecular biology have improved our understanding of not only cellular signaling and gene expression but also intercellular communication and long-distance integration of stress signals as a whole system during abiotic stress responses. Systematic knowledge of drought stress responses in whole plants can provide novel insights for agriculture and biotechnology. The critical information on drought stress resistance mechanisms merits further research for future crop innovation. For this purpose, phenomics analysis will be important in addition to other omics analyses to measure whole-plant phenotypes under stress conditions. Data science and imaging technology are powerful tools for the integration of these complex data to understand the complex systems underlying plant responses to environmental changes as a whole.

## Author Contributions

FT and KS designed work. FT, TK, KU, KY-S, and KS collected information from literature and wrote the manuscript. All authors contributed to the article and approved the submitted version.

## Conflict of Interest

The authors declare that the research was conducted in the absence of any commercial or financial relationships that could be construed as a potential conflict of interest.
