# Corrigendum: Drought Stress Responses and Resistance in Plants: From Cellular Responses to Long-Distance Intercellular Communication

**DOI:** 10.3389/fpls.2021.703149

**Published:** 2021-08-03

**Authors:** Fuminori Takahashi, Takashi Kuromori, Kaoru Urano, Kazuko Yamaguchi-Shinozaki, Kazuo Shinozaki

**Affiliations:** ^1^Gene Discovery Research Group, RIKEN Center for Sustainable Resource Science, Tsukuba, Japan; ^2^Gene Discovery Research Group, RIKEN Center for Sustainable Resource Science, Wako, Japan; ^3^Laboratory of Plant Molecular Physiology, Graduate School of Agricultural and Life Sciences, The University of Tokyo, Bunkyo-Ku, Japan

**Keywords:** drought stress, abscisic acid, peptides, transporters, protein kinases, metabolites, tissue-to-tissue communication

In the original article as published, the incorrect citation was included in the in two sentences in the *ABC Transporter Family* section (third paragraph). The correct citation should be “Pawela et al. (2019)”, not “Ding et al. (2008)”.

The published sentence “For example, in *Medicago truncatula*, MtABCG20 was reported as an ABA transporter (Ding et al., 2008).” should read “For example, in *Medicago truncatula*, MtABCG20 was reported as an ABA transporter (Pawela et al., 2019).”

The published sentence “Together, these data indicate that MtABCG20 is an ABA transporter that influences root morphology and nodulation in *Medicago truncatula* (Ding et al., 2008).” should read “Together, these data indicate that MtABCG20 is an ABA transporter that influences root morphology and nodulation in *Medicago truncatula* (Pawela et al., 2019).”

The authors apologize for this error and state that this does not change the scientific conclusions of the article in any way. The original article has been updated.

## Publisher's Note

All claims expressed in this article are solely those of the authors and do not necessarily represent those of their affiliated organizations, or those of the publisher, the editors and the reviewers. Any product that may be evaluated in this article, or claim that may be made by its manufacturer, is not guaranteed or endorsed by the publisher.
